# Prevalence of Radiographic Knee and Wrist Chondrocalcinosis in Adana Province, Türkiye: A Hospital-Based Retrospective Study

**DOI:** 10.5152/ArchRheumatol.2026.25248

**Published:** 2026-04-10

**Authors:** Erkan Kozanoğlu, Meryem Andırın, Övgü Bıçkıcı, Sevgül Köse

**Affiliations:** 1 Department of Physical Medicine and Rehabilitation, Çukurova University Faculty of Medicine, Adana, Türkiye; 2 Department of Physical Medicine and Rehabilitation, Division of Rheumatology, Çukurova University Faculty of Medicine, Adana, Türkiye; 3 Department of Radiology, Çukurova University Faculty of Medicine, Adana, Türkiye

**Keywords:** Chondrocalcinosis, knee, osteoarthritis, prevalence, wrist, X-ray

## Abstract

**Background/Aims:**

Multiple epidemiological studies in different ethnic groups demonstrate diverse results about the prevalence of chondrocalcinosis (CC), yet no definitive data are available concerning its frequency in most countries. Furthermore, the prevalence of CC in the Turkish population is not known to date. The aim of this study was to investigate the frequency of radiographic CC in the knee, wrist, or both joints in individuals aged ≥50 years from the hospital records of the last 5 years.

**Materials and Methods:**

In this retrospective study, radiographs of the knee and/or wrist taken at Çukurova University Faculty of Medicine Balcalı Hospital were reviewed by an experienced physiatrist, rheumatologist, and radiologist.

**Results:**

A total of 1818 radiographs from 772 individuals were included in the study. A total of 32 individuals exhibited calcification in at least 1 knee or wrist in total. Of these, 9.3% were male and 90.6% were female, with an overall radiographic prevalence of 4.1%. The prevalence of radiographic knee CC was found to be 4.1%, while that of wrist CC was 3.2%. Only 2 female patients exhibited CC in both the knee and wrist joints simultaneously. The frequency of CC in bilateral knee joints have been increased with age and no significant association was found regarding CC coexisting with other comorbidities except hyperparathyroidism.

**Conclusion:**

The prevalence of radiographically detected knee and wrist CC in the Turkish population has not yet been reported. These findings emphasize the need for clinical research that could increase awareness and advance knowledge of the relationship between CC and possible risk factors in Türkiye.

**Main Points**

- The radiographic prevalence of chondrocalcinosis (CC) in the study population was 4.1%.
- The prevalence of bilateral knee CC increases with age.
- The association of CC with knee osteoarthritis was not found to be significant.
- This study is among the few in the literature investigating the association of the prevalence of knee and wrist CC.
- This is the first study examining the association of knee and wrist CC in the Turkish population.

## Introduction

Calcium pyrophosphate crystal deposition disease (CPPD) is characterized by the deposition of calcium pyrophosphate crystals in cartilage and periarticular tissues and may present with asymptomatic, acute, or chronic clinical forms. Radiographic chondrocalcinosis (CC) refers to the calcification of hyaline cartilage and/or fibrocartilage and represents a common imaging manifestation associated with CPPD, although it is neither specific nor present in all affected patients with CPPD.^1^^,^^2^ The prevalence of CC increases with age and exhibits considerable variability across populations and study settings.^3^^,^^4^ A study conducted among US veterans reported the prevalence of CPPD as 5.2 per 1000 cases,^5^ but epidemiological data about the true prevalence of CC is limited.^4^Prevalence of knee or wrist CC has been reported in the literature as 5%-10%.[Bibr b6-ar-41-3-193]^6^Additionally, no major gender difference is reported in the literature. Macroscopic CPPD crystals were reported in 13% of cases in an autopsy study.^7^ This variability in prevalence may be influenced by differences in age distribution, imaging indications, joint regions evaluated, and underlying metabolic or degenerative conditions. Notably, CC most frequently affects the knee joint, although involvement of the wrist and other joints has also been described.^4^

Despite the growing recognition of CPPD and CC in aging populations, data regarding the prevalence of radiographic CC remain scarce in many countries. In particular, epidemiological information on CC frequency in the Turkish population is lacking. Understanding regional prevalence may contribute to a better characterization of disease burden and facilitate comparisons with existing international data.

The primary aim of this study was to investigate the prevalence of sporadic radiographic CC in knee and/or wrist radiographs obtained from adults at a tertiary care center over an approximately 5-year period. The secondary aim of the study was to evaluate the distribution of CC according to joint involvement and demographic characteristics.

## Materials and Methods

### Study Population

In this retrospective study, knee and/or wrist radiographs obtained between June 2018 and January 2024 at Çukurova University Faculty of Medicine Balcalı Hospital. All radiographs were independently reviewed by an experienced physiatrist and a rheumatologist. The assessments were performed with access to basic radiographic indications, as this was a retrospective study. In cases of disagreement, images were re-evaluated by a physiatrist, rheumatologist, and radiologist jointly, and thereafter, a consensus decision was reached. This consensus-based approach was used to reduce inter-observer variability. Radiographs from the last 5 years were included to ensure homogeneity in imaging quality and radiographic techniques and to reflect contemporary clinical practice as well as to provide accurate patient records. Knee and wrist joints were selected because they are the most frequently evaluated joints for radiographic CC and are commonly involved in CPPD, allowing for a standardized and comparable assessment of the prevalence.

The inclusion criteria were defined as being over 50 years and having undergone knee and/or wrist radiographic examination for non-traumatic reasons who also had obtainable medical records. Radiologic examination for patients in the emergency department was excluded.

Radiographs that had been performed for traumatic reasons were excluded based on both radiographic findings (fracture or dislocation) and review of clinical documentation indicating trauma-related imaging.

Radiographs were obtained in standard anteroposterior and lateral projections for the knee and posteroanterior projection for the wrist. Chondrocalcinosis was evaluated based on the presence of linear or punctate calcifications in hyaline and/or fibrocartilage, and the areas of involvement were recorded as the fibrocartilage of the medial and lateral menisci, femoral condyles, tibial plateau, and patellofemoral joint in the knee joint, and the radiocarpal and intercarpal joints, primarily the triangular fibrocartilage and the area around the scapholunate ligament in the wrist.^8^ The same radiographs were evaluated based on radiographic criteria such as the presence of osteoarthritis (OA), joint space narrowing, marginal osteophyte formation, subchondral sclerosis, and cyst development; the tibiofemoral and patellofemoral compartments in the knee and the radiocarpal and intercarpal joints in the wrist were considered.^9^

X-rays were divided into 3 groups: individuals with only unilateral/bilateral knee radiographs, individuals with only unilateral/bilateral wrist radiographs, and individuals with unilateral/bilateral knee and wrist radiographs. The following data were recorded for each patient: age, gender, comorbidities, location of CC involvement (unilateral/bilateral, fibrous/hyaline cartilage), presence of concomitant OA, and stage of OA according to the Kellgren–Lawrence classification for knee X-rays. Wrist radiographs were assessed based solely on plain radiographic findings, and cases showing typical features of CC were accepted as CC. No additional imaging or systematic evaluation for differential diagnoses such as sesamoid bones or accessory ossicles was performed.

Data regarding comorbidities such as diabetes mellitus, hyperparathyroidism, hypothyroidism, spondyloarthropathy, and rheumatoid arthritis were retrospectively extracted from the hospital information system by reviewing department admissions, long-term medication reports, and relevant laboratory findings available in electronic medical records (Tables 1 and 2).

The present study was conducted in accordance with the Declaration of Helsinki and was approved by the Ethics Committee of the Çukurova University Faculty of Medicine (Decision No: 23 of meeting No: 140 dated January 4, 2024). This study was designed as a retrospective X-ray evaluation of files in the radiology department. Therefore, informed consent was not obtained.

### Radiography

All radiographs were performed using General Electric (GE), Proteus and GE, Silhouette conventional radiographic systems and GE, Optima digital radiographic system. Examinations were reviewed using the Enlil Picture Archiving and Communication Systems and workstations. Image manipulation including window and level adjustment and magnification was applied as needed to optimize image interpretation.

### Statistical Analysis

Categorical measurements are represented by numbers and percentages, while numerical measurements are represented by the mean and standard deviation. The Shapiro–Wilk test was used to confirm the normality of distribution for continuous variables. To compare categorical variables between groups, the Pearson chi-Square test or Fisher’s exact test was used, depending on whether the expected value problem arose. One-way analysis of variance was used to compare continuous variables between the age groups. If a significant difference was found between groups, a pairwise comparison method such as Tukey’s test was used. All analyses were performed using the IBM SPSS Statistics, version 20 statistical software package (IBM SPSS Corp.; Armonk, NY, USA). The statistical significance level for all tests was set at 0.05.

## Results

**Figure 1.**
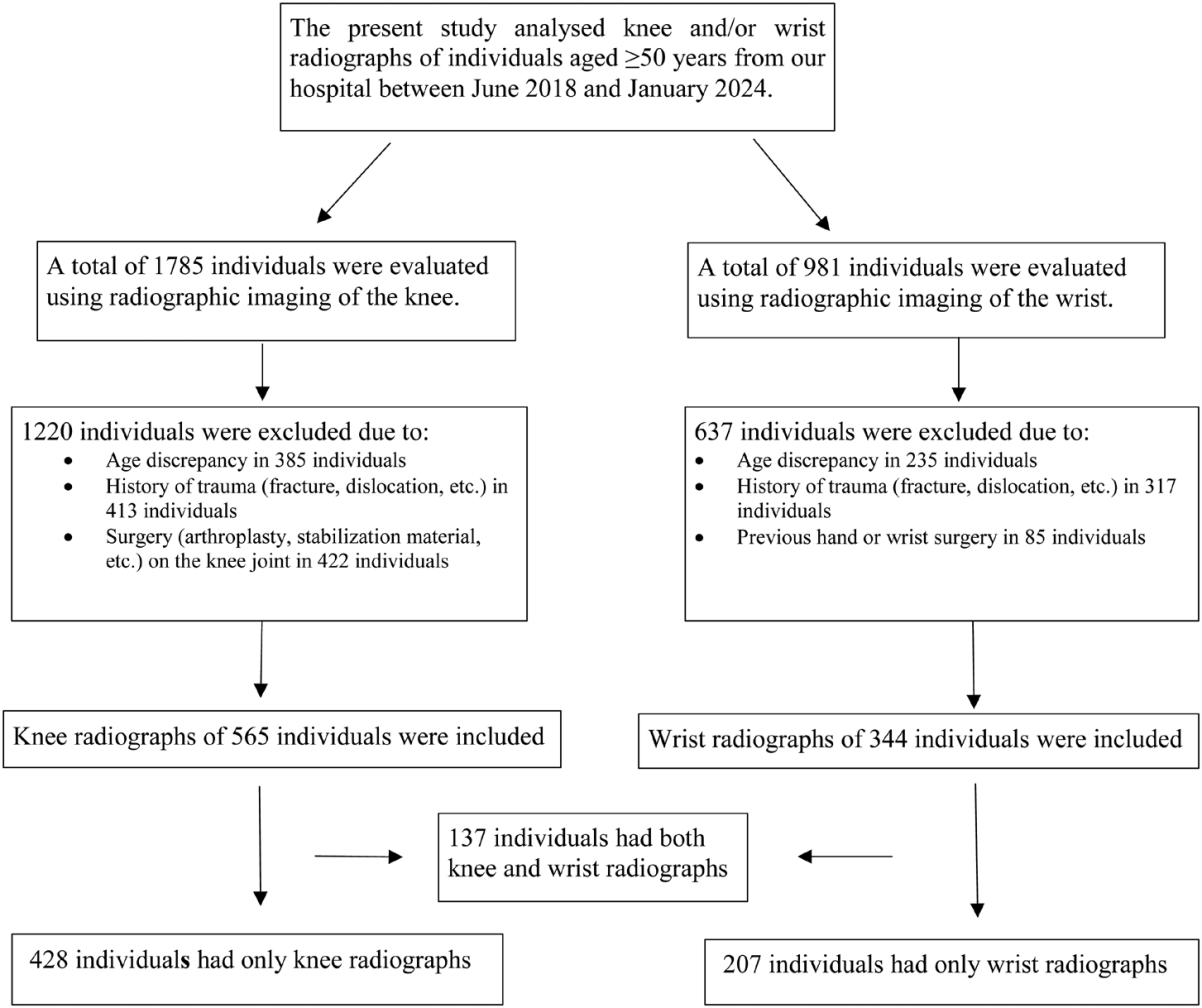
Flow chart of the study.

A total of 772 individuals were included in the study. The flow diagram demonstrating study inclusion and exclusion is presented in Figure 1. Of the individuals, 550 (71.2%) were female and 222 (28.8%) male. A total of 32 individuals exhibited calcification in at least 1 knee or wrist joint in total. Of these, 9.3% were male and 90.6% were female, with an overall prevalence of 4.1%. The knee joint was found to be more frequently affected than the wrist joint (67.6% and 32.3%, respectively). When the prevalence was considered by gender, the results indicated that 5.2% of women had been diagnosed with CC, compared to 1.3% of men.

### Radiographic Findings in Patients with Knee Radiographs

When individuals with knee radiographs were analyzed, CC was present in at least 1 knee joint in 4.1% of individuals (23/565). The distribution of knee joint involvement among individuals with CC involvement was as follows: 43.5% had involvement of the right knee, 26.1% had involvement of the left knee, and 30.4% had involvement of both knees. In 10 out of 23 cases (43.5%), fibrous cartilage involvement was found, while in 13 out of 23 cases (56.5%) hyaline cartilage involvement was observed. The present study revealed that all individuals with CC in the knee joint were female, a result that achieved statistical significance (*P* = .003). The prevalence of CC increased with advancing age, except for the 60-69 age group. The frequency of CC in bilateral knee joints have been increased with age (*P* = .040). The presence of bilateral CC was 28.6% in women aged 50-69 years; however, this rate increased to 71.4% in women over the age of 70 years. No significant association was found regarding the presence of CC with other comorbidities except hyperparathyroidism (*P* = .041) (Table 1). All patients with knee CC had radiographic evidence of OA, whereas OA was also common among patients without CC. However, the association between knee OA and CC was not statistically significant. The relationship of OA with knee and wrist CC is presented in Table 3.

### Radiographic Findings in Patients with Wrist Radiographs

Wrist radiographs from individuals aged ≥50 years (245 female and 99 male) were analyzed, as detailed in Figure 1. Chondrocalcinosis was found in 3.2% of individuals at least 1 wrist joint. Of these, 3 individuals (27.3%) were male and 8 individuals (72.7%) were female. Among the individuals with CC involvement, 6 had right wrist involvement (54.5%), 4 had left wrist involvement (36.4%), and 1 had bilateral wrist involvement (9.1%). Of the individuals with CC involvement, 1 out of 11 (9.1%) had fibrous cartilage involvement, while 10 out of 11 (90.9%) had hyaline cartilage involvement. Among male individuals, CC was found in the right hand in 1 out of 3 individuals and in the left hand in 2 out of 3 individuals. For female individuals, CC was found in 5 out of 8 individuals in the right hand (62.5%), in 2 out of 8 individuals in the left hand (25%), and in 1 out of 8 individuals in both hands (12.5%) (Table 1). The relationship between the presence of CC in the wrist and wrist OA was statistically significant (*P* < .001) (Table 3).

### Radiographic Findings in Patients with Both Knee and Wrist Radiographs

**Figure 2.**
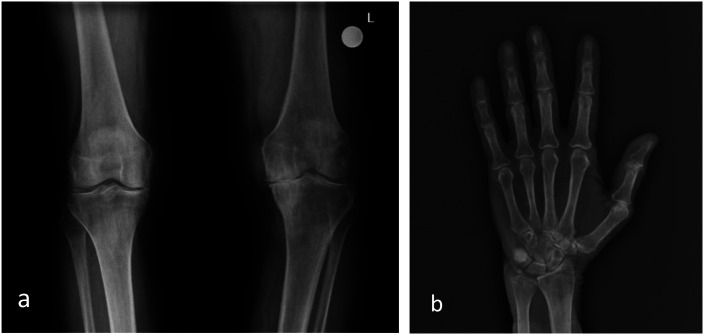
Deposition of CCPD crystals in knee and wrist joints: (a) meniscal cartilage involvement; (b) triangular fibrocartilage involvement.

Radiographic findings of both knee and wrist radiographs of 137 individuals (109 females, 28 males) were also assessed. It was observed that only 2 individuals (1.4%) exhibited simultaneous CC in both the wrist and knee as demonstrated in Figure 2. One individual exhibited CC involvement in both knees and wrists bilaterally, while the other individual had CC involvement only in the right knee and right wrist. Demographic characteristics of individuals both with knee and wrist CC are shown in Table 2.

## Discussion

The primary objective of this study was to ascertain the frequency of CC in knee and wrist X-rays ordered for various reasons in the center. A total of 1818 X-rays were examined, revealing a prevalence of 4.1% for CC, a condition that increases with age and is more prevalent in knee joints than in wrists. Notably, this is the first study conducted in the southern part of Türkiye aimed at investigating the overall prevalence of CC in a relatively large population.

When compared with previous studies, the overall frequency of 4.1% observed in the study is largely consistent with findings from different international settings. For instance, a study conducted in Iran, which analyzed 600 X-rays of the knees, shoulders, and wrists of patients over the age of 50, reported a prevalence of 3.8%. Similar to the findings, that study also demonstrated that the frequency of CC increases with age.^^10^^ Likewise, a study performed at a Mexican tertiary care center reported an overall prevalence of 3% among 3350 knee and wrist radiographs of patients aged over 50 years.^11^

However, some studies have reported higher prevalence rates. For example, a study from Italy examining 3099 knee-pelvic radiographs revealed a prevalence of 10%, with the knee being the most commonly affected anatomical region. The authors noted that this higher prevalence aligns with other European studies.^12^ Similarly, a study from the UK in 2003 reported a crude prevalence of 7%, which was subsequently adjusted to 4.5% after controlling for factors such as age, sex, and knee pain frequency.^13^

These variations in prevalence among different studies may be attributed to a number of factors, including ethnic diversity, differences in study populations, sample sizes, and demographic characteristics such as age and sex. Despite these variations, the overall prevalence observed in the study appears consistent with the global literature.

In addition, Abishek et al conducted a large-scale study evaluating the distribution of CC using radiographs of the knee, pelvis, and hand from 3170 individuals. Their results demonstrated that the prevalence of CC at any site was 13.7%, with the knee being the most commonly affected joint (8%), followed by the wrist (6.9%), hip (5%), symphysis pubis (3.6%), and carpometacarpal joint (1.5%). Within the knee, CC was more frequently observed in the lateral tibiofemoral compartment compared to the medial, and it was more common in the menisci than in the hyaline cartilage. Interestingly, the study found no evidence suggesting that meniscal or hyaline cartilage CC predisposes to fibrocartilage CC in distant joints.^14^

In addition, a cohort study from South Korea investigating the incidence and risk factors of knee CC reported a crude incidence of 3.19 per 1000 person-years and a cumulative incidence of 2.7%. This study highlighted advanced age (>55 years) and elevated hemoglobin A1c levels as significant risk factors for knee CC. Notably, the highest incidence was reported in individuals aged 60-69. However, other factors such as gender, body mass index, hypertension, and alcohol consumption did not show significant associations with the development of knee CC. Furthermore, while radiographic knee OA was associated with an increased risk of CC, this association did not reach statistical significance.^15^ Consistent with this, the study also revealed a non-significant difference in CC prevalence between individuals with and without knee OA.

Chondrocalcinosis has been reported in association with various endocrine, metabolic, and rheumatic conditions, including hyperparathyroidism, hemochromatosis, and inflammatory arthritides like rheumatoid arthritis.^16^ In contrast, findings regarding other metabolic conditions like hypertension, diabetes mellitus, and medications remain conflicting in the literature, which may partly be explained by differences in disease definitions and analytic approaches across studies.^17^ In the present hospital-based retrospective study, CC was more frequently observed in patients with hyperparathyroidism, and a statistically significant increase in knee CC was noted in this subgroup. However, this observation was based on a very small number of patients, including a single individual with hyperparathyroidism and should therefore be interpreted with caution. Furthermore, the retrospective radiographic design, absence of clinical evaluation, and hospital-based nature of the study limit causal inference, and regional characteristics of the study population may also have influenced the observed distribution of comorbidities. Accordingly, these findings should be considered descriptive rather than indicative of a definitive association.

Although the association between CC and sex varies across studies, the majority of epidemiological investigations report a higher prevalence in women compared with men. Overall, women appear to be affected approximately 1.4 times more frequently than men.^18^ For instance, population-based data from Italy demonstrated a prevalence of 12.8% in women vs. 7% in men.^^12^^ Marked sex differences have also been described in specific anatomical sites; CC of the temporomandibular joint is substantially more common in women, with reported rates of up to 17% compared with approximately 1% in men.^19^ Similarly, female predominance has been observed in commonly involved joints such as the knee and wrist, whereas no clear sex predilection has been identified for pelvic involvement.^20^

In the context of the findings, the significantly higher prevalence of knee CC observed in women (*P* = .003) is concordant with the literature which supported the hypothesis that sex-specific factors (potentially including hormonal influences on cartilage metabolism, differential OA burden, and age-related biomechanical changes) might contribute to the pathogenesis and anatomical expression of CPPD disease. These observations highlight the importance of considering sex as a relevant variable in both epidemiological studies and mechanistic investigations of CC.

Nevertheless, the relationship between CC and OA remains a topic of ongoing research. While previous meta-analyses have suggested an association between knee CC and knee OA, findings across different joints are inconsistent.^21^ In the present study, CC was more frequently observed in patients with wrist OA, whereas no significant association in CC was observed between patients with and without knee OA. These findings suggest that the distribution of CC in relation to OA may vary according to joint involvement and study population.

In the cohort, OA was not detected in the non-CC wrist group and it might likely affect the statistical results. But, wrist OA has been reported to have a relatively low prevalence compared with other joint regions. For example, data from the Framingham Osteoarthritis Study demonstrated a prevalence of 1.7% in men and 1.0% in women, with similarly low incidence rates during follow-up. In addition, previous wrist fractures were common among wrists with OA in that population.^^22^^ Since trauma-related radiographs were excluded in the present study and the number of male patients was lower than female patients, the absence of wrist OA in the non-CC group might reflect the characteristics of this selected hospital-based sample rather than a true clinical absence. Therefore, this finding should be interpreted cautiously and considered descriptive rather than indicative of a definitive clinical relationship.

The present study has certain limitations. Firstly, being a single-center study, the findings may not be generalizable to the entire Turkish population. Secondly, as this was a hospital-based retrospective radiographic study, the study population consisted only of individuals who underwent imaging for various clinical indications, which may have introduced sampling bias. Therefore, the reported frequency reflects the characteristics of this selected population rather than true population-based prevalence. Thirdly, the retrospective radiographic nature of the study limited access to clinical parameters such as body mass index, medication use, and laboratory data, which restricted the ability to comprehensively evaluate potential contributing factors. Due to the low prevalence of CC in the study population, the number of CC cases was insufficient to support advanced multivariable analyses. Consequently, the findings are primarily based on univariable, age-focused analyses and should be interpreted with caution.

In conclusion, there are a few studies in the literature evaluating the prevalence of radiographic knee and wrist CC together. To the best current knowledge, there is no study about the prevalence of radiographic knee and wrist CC in Türkiye. The prevalence of knee CC was found to be 4.1%, while that of wrist CC was 3.2%. Notably, only 2 female patients exhibited CC in both the knee and wrist joints simultaneously. The findings demonstrated that the prevalence of bilateral knee CC increases with age. Although the prevalence of CC was higher among patients with knee OA, this difference was not statistically significant. Conversely, CC was more frequently observed in patients with wrist OA. These findings emphasizes the need for clinical research that could increase awareness and advance knowledge of the relationship between CC and possible risk factors in Türkiye.

### Data Availability Statement:

The data that support the findings of this study are available on request from the corresponding author.

### Artificial Intelligence Usage Statement:

The authors declared that no Artificial Intelligence tool was used in the preparation of the manuscript.

### Ethics Committee Approval:

Ethical committee approval was received from the Çukurova University Faculty of Medicine Ethics Committee (Decision No: 23 of meeting No: 140 ; Date: January 4, 2024).

### Informed Consent:

N/A.

### Peer-review:

Externally peer-reviewed.

### Acknowledgment:

The authors would like to express their gratitude to Associate Professor İlker Ünal for his invaluable support in this study.

### Author Contributions:

Concept – E.K.; Design – E.K., M.A.; Supervision – E.K., S.K.; Resources – M.A., Ö.B.; Materials – M.A., Ö.B.; Data Collection and/or Processing – E.K., M.A., Ö.B.; Analysis and/or Interpretation – E.K., M.A., Ö.B., S.K.; Literature Search – M.A., Ö.B.; Writing – E.K., M.A., Ö.B.; Critical Review – E.K., S.K.

### Declaration of Interests:

The authors have no conflicts of interest to declare.

### Funding:

The authors declare that this study received no financial support.

## Figures and Tables

**Table 1.** Demographic Characteristics of Individuals with Knee or Wrist Chondrocalcinosis

**Variables** ​	**Knee Chondrocalcinosis**		*P*​	**Wrist Chondrocalcinosis**		*P*​
	**Yes (n = 23)**	**No (n = 542)**		**Yes (n = 11)**	**No (n = 333)**	
Age (years)	​	​	​	​	​	​
Mean	62.6 ± 11.7	63.4 ± 8.4	*.737	63.7 ± 8.1	61.6 ± 8.06	*.398
50-59	12 (5.5)	205 (94.5)	​	5 (3.1)	155 (96.9)	​
60-69	2 (1)	198 (99)	*.040	2 (1.6)	120 (98.4)	*.285
70-79	7 (5.5)	120 (94.5)	​	4 (7.1)	52 (92.9)	​
≥80	2 (9.5)	19 (90.5)	​	0 (0)	6 (100)	​
Sex	​	​	​	​	​	​
Male	0 (0)	151 (100)	***.003	3 (3)	96 (97)	**1.00
Female	23(5.6)	391 (94.4)	​	8 (3.3)	237 (96.7)	​
Comorbidities	​	​	​	​	​	​
Hypertension	8 (3.9)	197 (96.1)	***.879	2 (2)	97 (98)	**.736
Diabetes	1 (1.1)	86 (98.9)	**.232	0 (0)	33 (100)	**.609
Hypothyroidism	1 (7.1)	13 (92.9)	**.445	1 (20)	4 (80)	**.151
Hyperparathyroidism	1 (100)	0 (0)	**.041	1 (50)	1 (50)	**.063
Spondyloarthropathy	0 (0)	20 (100)	**1.00	0 (0)	14 (14)	**1.00
Rheumatoid arthritis	4 (8.9)	41 (91.1)	**.101	3 (6.7)	42 (93.3)	**.163

*t-test for independent groups.

**Fisher’s exact test.

***Pearson chi-square test.

**Table 2.** Demographic Characteristics of Individuals Both with Knee and Wrist Chondrocalcinosis

**Variables** ​	**Knee + Wrist Chondrocalcinosis**		*P*​
	**Yes (n = 2)**	**No (n = 135)**	
Age (years)	70.6 ± 9.3	62.6 ± 7.5	*<.005
Sex	​	​	​
Male	0 (0)	28 (100)	​
Female	2 (1.8)	107 (98.2)	​
Comorbidities	​	​	​
Hypertension	1 (2)	48 (98)	​
Diabetes	0 (0)	13 (100)	​
Hypothyroidism	1 (33.3)	2 (66.7)	​
Hyperparathyroidism	1 (100)	0 (0)	​
Spondyloarthropathy	0 (0)	11 (100)	​
Rheumatoid arthritis	0 (0)	17 (100)	​

*t-test for independent groups.

**Table 3.** Association of Osteoarthritis and Chondrocalcinosis

**Association of Osteoarthritis**​	**Knee Chondrocalcinosis**		*P*​	**Wrist Chondrocalcinosis**		*P*​
	**Yes (n = 23)**	**No (n = 542)**		**Yes (n = 11)**	**No (n = 333)**	
No	0 (0)	4 (0.7)	**0.776	9 (81.8)	333 (100)	**<.001
Yes	23 (100)	538 (99.3)	​	2 (18.2)	0 (0)	​
Grade-1	0 (0)	7 (1.3)	​	​	​	​
Grade-2	4 (17.4)	142 (26.4)	​	​	​	​
Grade-3	15 (65.2)	296 (55.1)	​	​	​	​
Grade-4	4 (17.4)	93 (17.2)	​	​	​	​

**Fisher’s exact test. Values highlighted in bold indicate statistical significance.
